# RE-AIM Framework Guides Comprehensive Mixed-Methods Evaluation of GRECC Interprofessional 5Ms Core Curriculum

**DOI:** 10.1093/geroni/igaf122.740

**Published:** 2025-12-31

**Authors:** Kathryn Nearing, Huai Cheng, Elizabeth Chapman, Katharina Echt, Iriana Hammel, Beth Hogans, Sumi Misra, Marianne Shaughnessy

**Affiliations:** University of Colorado Anschutz Medical Campus, Denver, Colorado, United States; Minneapolis VA Hospital, Minneapolis, Minnesota, United States; William S. Middleton Memorial Veterans’ Hospital, Madison, Wisconsin, United States; U.S. Department of Veterans Affairs, Brookhaven, Georgia, United States; Miami VA Medical Center, Miami, Florida, United States; Johns Hopkins School of Medicine, Baltimore, Maryland, United States; Vanderbilt University Medical Center, Nashville, Tennessee, United States; Veterans Health Administration, MIDDLE RIVER, Maryland, United States

## Abstract

The national evaluation of the interprofessional Geriatric 5Ms core curriculum is guided by the RE-AIM framework from implementation science. RE-AIM stands for reach (who accesses and completes the curriculum), effectiveness (the degree to which the curriculum increases awareness, knowledge, self-efficacy and intentions to apply new learning in practice), adoption (uptake of the curriculum by the national GRECC network), implementation (how the curriculum is integrated into other training requirements locally), and maintenance (sustained use of the core curriculum by GRECC health professions training programs). RE-AIM focuses our comprehensive evaluation at multiple levels salient to the national roll-out of a new curriculum across varied local contexts and different configurations of associated health professions training programs. RE-AIM directs us to assess the national reach of the curriculum to address current gaps/needs for geriatric/gerontologic education, while also assessing facilitators and barriers to local adoption and implementation. Key lessons learned can be applied to promote dissemination. This individual abstract session will provide an overview of the mixed-methods evaluation of the new core curriculum, including the national learning management system available to ensure all GRECC health professions trainees are assigned and complete the curriculum as part of their learning plans and receive a standardized evaluation to assess key outcomes such as satisfaction with the quality of content and delivery, gains in core competencies, and intentions to apply new learning in practice. We will conclude by sharing early evaluation findings, including emerging implementation models from the field.

